# The Role of a “Conservative” Resection Strategy After Neoadjuvant Treatment for Borderline/Locally Advanced PDAC with Arterial Involvement: A Single-Centre Retrospective Observational Study

**DOI:** 10.3390/cancers18050830

**Published:** 2026-03-04

**Authors:** Roberta Vella, Elisa Bannone, Alessandro Giardino, Isabella Frigerio, Martina Guerra, Erica Pizzocaro, Laura Bignotto, Filippo Scopelliti, Paolo Regi, Camillo Aliberti, Guido Martignoni, Roberto Girelli, Marcello Lino, Paolo Pederzoli, Giovanni Butturini

**Affiliations:** 1 HPB Surgery, Pederzoli Hospital, 37019 Peschiera del Garda, Italy; roberta.vella02@unipa.it (R.V.); isabella.frigerio@ospedalepederzoli.it (I.F.); martina.guerr@gmail.com (M.G.); laura.bignotto@ospedalepederzoli.it (L.B.);; 2 Department of Precision Medicine in Medical, Surgical, and Critical Care, (Me.Pre.C.C.), University of Palermo, 90133 Palermo, Italy; 3 PhD School of Applied Medical-Surgical Sciences, University Tor Vergata, 00133 Rome, Italy; 4 Unit of Radiology, Pederzoli Hospital, 37019 Peschiera del Garda, Italy; 5 Pathology Unit, Pederzoli Hospital, 37109 Peschiera del Garda, Italy; 6 Vascular and Endovascular Unit, Department of Surgery, Pederzoli Hospital, 37019 Peschiera del Garda, Italy

**Keywords:** pancreatic adenocarcinoma, locally advanced pancreatic cancer, conservative surgery, folfirinox, arterial resection, arterial divestment

## Abstract

Background: Recent advances in multimodal therapies have increased the potential for resectability of borderline resectable and locally advanced pancreatic ductal adenocarcinoma (PDAC). We herein describe the conservative resection strategy adopted at our institution and the oncological outcomes of patients with PDAC and arterial involvement. Methods: This retrospective single-centre study included patients diagnosed with PDAC and radiologic evidence of arterial involvement who underwent surgical exploration between January 2014 and June 2024. All patients received induction chemotherapy (±radiotherapy). Survival outcomes were analyzed using the Kaplan–Meier and Cox proportional hazards models. Logistic regression analyses were used to identify predictors of resectability and recurrence. Results: A total of 76 patients were included: 59 underwent pancreatic resection with arterial divestment (AD) in case of persistent arterial involvement and 17 were deemed unresectable at laparotomy. Neoadjuvant folfirinox was significantly associated with increased odds of resection (HR = 3.23, 95% CI: 1.59–9.90, *p* = 0.040). Median overall survival from diagnosis was 33 months (29–39) in resected patients and 26 months (16–29) in non-resected patients (*p* = 0.0176). Surgical resection and Ca 19,9 normalization after induction therapy were associated with reduced mortality risk (HR = 0.38, 95% CI: 0.19–0.75, *p* = 0.005 and HR = 0.56, 95% CI: 0.35–0.88, *p* = 0.014, respectively). Conclusions: Despite a limited sample size and retrospective nature, these findings highlight the value of multimodal strategies in managing PDAC with arterial involvement. AD represents a valuable technique associated with acceptable outcomes in selected patients. Future interventional prospective studies are needed to optimize patient selection and validate the prognostic role of extended surgical procedures.

## Simple Summary

A conservative surgical approach with arterial divestment after neoadjuvant therapy in LAPC is feasible and associated with survival benefit, offering acceptable oncological outcomes. Careful patient selection remains essential given imaging limitations and the biological heterogeneity of the disease.

## 1. Introduction

Pancreatic ductal adenocarcinoma (PDAC) is one of the leading causes of cancer deaths worldwide with a dismal prognosis and increasing incidence [1,2]. Since the introduction of effective multi-chemotherapy regimens, such as fluorouracil plus leucovorin plus irinotecan plus oxaliplatin (folfirinox) and gemcitabine with nab-paclitaxel [3,4], in the neoadjuvant setting, surgical resection for PDAC has gone beyond the limits of standard indications, including patients with initially unresectable tumours [5,6,7]. Nevertheless, no definitive consensus exists regarding the extent of arterial involvement after neoadjuvant therapies (NATs) or the efficacy of extended surgical resections, resulting in suboptimal patient stratification.

Following the pioneering work of Fortner et al. [8] on arterial resection (AR) during pancreatectomies in 1973, the concept has undergone a long yet gradual evolution. Over the decades, surgical approaches have expanded to include patients with initially unresectable PDAC involving arterial structures.

Traditionally, the willingness to reach an R0 resection has pushed surgeons to perform AR combined with pancreatectomy as the primary approach for treating locally advanced PDAC (LAPC), particularly after a favourable response to neoadjuvant therapy [9,10,11]. Despite historically poor outcomes [12], centralization and improvement in technical skills have led to a significant reduction in postoperative morbidity and mortality in high-volume centres [13,14,15,16,17,18]. However, the prognostic value of this demanding surgery is still questioned, particularly given that histological studies have shown that a relevant proportion (55–60%) of resected arterial specimens do not harbour true tumour infiltration [13,14,15].

Considering the pancreatic cancer biology and its neurotropism [19], which accounts for the high rate of perineural invasion and dismal prognosis, Hackert et al. introduced the concept of extensive clearance of the perivascular soft tissue (the so-called “Triangle Operation”) as part of a pancreatic resection for reducing the rate of local recurrence after potentially curative resection [20]. This oncologic principle has been further reinforced by growing evidence distinguishing true arterial wall invasion from mere arterial involvement without histological infiltration—particularly in the post-neoadjuvant setting—highlighting its critical prognostic implications [21]. These insights have fostered the development of conservative surgical strategies, including periarterial divestment, periadventitial dissection, and sub-adventitial divestment, that focus on preserving arterial integrity while achieving curative resection [22,23]. Miao et al. [24] first coined the term “arterial divestment” (AD) for describing the standardized technique of sub-adventitial divestment which separates the tumour from the artery by dissecting into the plane between the external elastic of tunica adventitia and the tunica media of the involved artery. Since then, several authors have reported their experiences and strategies for effectively stripping the encasing lymphatic and perineural tissue off the artery to obtain a complete oncologic resection [22,25,26,27,28,29]. In this context, some authors suggest performing frozen sections of the suspicious periarterial tissue to guide intraoperative arterial management [22,30].

Recently, the REDISCOVER guidelines have attempted to shed light on the growing role of extended pancreatic surgery, including AR and/or AD, yet no definitive agreement has been reached [31].

Concurrently, radiographic criteria for PDAC staging—particularly regarding arterial involvement—have evolved, with the extent of circumferential contact with the vessel wall (e.g., 180° threshold) now used to distinguish borderline resectable from locally advanced disease [32,33]. However, studies reveal inconsistencies between radiological evaluation of vascular infiltration, especially after NAT, and surgical outcomes, as circumferential involvement does not always correlate with histological invasion [34,35].

The primary aim of this study was to evaluate overall survival in patients with borderline resectable or LAPC undergoing conservative resection after neoadjuvant therapy in a high-volume centre. Secondary aims included identifying predictors of resectability and recurrence in this cohort.

## 2. Materials and Methods

### 2.1. Patient Cohort and Data Collection

This single-centre retrospective study included all consecutive patients with a histological diagnosis of ductal adenocarcinoma of the pancreas and radiological evidence of arterial infiltration (common hepatic artery, superior mesenteric artery, celiac trunk), eventually associated with venous infiltration, who received chemotherapy and/or radiotherapy followed by surgical exploration from January 2014 to June 2024.

Data were collected from an electronic prospectively maintained database and retrospectively analyzed.

Standard demographic data, induction chemotherapy regimen and radiotherapy, radiological data including resectability status at diagnosis and radiological response after induction therapies, and CA 19,9 levels before and after chemotherapy were collected. Surgical procedure was also recorded as pancreatoduodenectomy (PD), distal pancreatectomy (DP), total pancreatectomy (TP), or explorative laparotomy (EP). Perivascular biopsies and AD were respectively performed to assess persistent tumoural tissue or achieve arterial R0 resection when feasible. Pathological data encompassed tumour size, nodal status, tumour grade and R status [33]. Postoperative data included complications, graded according to the Clavien–Dindo (CD) classification [36], length of hospital stay, reintervention, in-hospital death, and failure to rescue rate (FTR). Follow-up and survival data were collected via phone interviews and/or clinical consultations. Minimum follow-up was 12 months. Written informed consent was obtained from all subjects for the use of their clinical data in clinical research.

### 2.2. Inclusion Criteria

Only patients with available preoperative imaging were selected. Preoperative imaging included chest and computed tomography (CT) with pancreas protocol, and contrast-enhanced magnetic resonance (MRI). All images were reviewed independently by one expert radiologist (CA) and one expert surgeon (GB).

The resectability status at diagnosis was defined according to the National Comprehensive Cancer Network (NCCN) resectability criteria in force at the time of surgery [33,37]. After NAT, all patients underwent radiological restaging and the arterial involvement was defined according to the interface between the tumour and the artery (T/A) as follows: abutment if contact is 180° or less and encasement if T/A interface was greater than 180°. All patients underwent multidisciplinary evaluation and surgery was proposed based on the following criteria: (1) reduction in CA 19,9 value, (2) absence of tumour progression, (3) absence of metastatic disease at restaging, (4) good performance status (Karnofsky 80% or more).

All patients included in the study had undergone a staging laparoscopy, which was negative for peritoneal dissemination or for peritoneal cytology. Patients with occult metastatic disease (macroscopic peritoneal disease or positive cytology) were excluded from the surgical cohort analyzed in this study.

### 2.3. Surgical Procedure

Superior mesenteric artery (SMA) resections are not performed in our centre, as per our institutional policy, differently from celiac axis (CA) resection for body–tail tumours with persistent infiltration after NAT.

Artery first approach [38] was routinely performed: in the presence of suspicious periarterial tissue, whenever the operating surgeon believed that it could have relevant importance in decision-making, peri-adventitial biopsies were sent for intraoperative histology. Surgical resection was aborted because of intraoperative evidence of non-resectability, as determined by the operating surgeon, either for positivity on the frozen section of unresectable tissue or for other peculiar situations of technically infeasible resections. In those cases, palliative bypass procedures were performed when needed. Additionally, radiofrequency ablation (RFA) procedures were performed in this group of patients from January 2014 to August 2016 as part of a multicentre randomized controlled trial [39]. Venous resections were performed when necessary to achieve R0. All venous resections were type III according to ISGPS classification [40]. All surgical procedures were performed by seven experienced pancreatic surgeons (GB, PP, RG, AG, IF, PR, FS). At the surgeon’s preference, the assistance of an expert vascular surgeon (ML) was guaranteed.

### 2.4. Survival Outcomes

Overall survival (OS) from diagnosis was defined as the time from diagnosis to death. Survival from surgery was defined as the time from the date of surgery until death. Recurrence-free survival (RFS) was defined as the time between resection and reappearance of disease; post-recurrence survival (PRS) was defined as survival from recurrence to death. Early recurrence was defined as recurrence within 6 months after resection [41].

### 2.5. Statistical Analysis

The normality of data was assessed through the Shapiro–Wilk test. Whenever normality was not respected, non-parametric tests were used. Continuous data were expressed as the median and interquartile range and compared using the two-sample *t*-test; categorical variables were presented as numbers and percentages and compared using Chi-square or Fisher’s exact test, as appropriate. Diagnostic accuracy analysis was performed by comparing CT-based predictions of resectability and perivascular infiltration with the corresponding pathologic outcomes and evaluating the area under the receiver operating characteristic (ROC) curve (AUC). Univariate and multivariate logistic regression analyses were performed to estimate adjusted odds of the outcomes of interest. Survival curves were created using the Kaplan–Meier (KM) method and analyzed using the log-rank test. Cox proportional hazards models were used for evaluating related hazard ratios. Conditional survival was calculated as the probability of survival for x additional years (CSx) given y years of accumulated survival. All statistical analyses were performed using STATA Software (ed.17). Differences were considered statistically significant at *p* < 0.05.

The manuscript was prepared in accordance with the STROBE guidelines for reporting observational cohort studies [42].

## 3. Results

### 3.1. Baseline Characteristics

Table 1 summarizes baseline and restaging data for the 76 included patients with borderline resectable (BR) or locally advanced pancreatic cancer (LAPC) and arterial involvement who underwent surgical exploration after NAT. All patients received neoadjuvant chemotherapy (folfirinox 60.53%, Gemcitabine alone 6.8%, Gem + Nab-paclitaxel 23.68%, folfirinox + gem-nab paclitaxel 1.32%, gemox 2.63%, PAX-G 2.63%), and 36/76 (47.37%) underwent induction chemo-radiotherapy. A total of 29 out of 36 patients (80.5%) who had a full course of chemo- and radiotherapy were resected versus 30 out of 40 patients (75%) who had only chemotherapy as induction treatment: the difference is not statistically significant (*p* > 0.05). After NAT, 9/76 (11.84%) patients showed no residual arterial contact, while arterial abutment persisted in 48/76 patients (63.16%) and encasement in 19/76 patients (25%), with SMA as the most frequently involved (40/67, 59.7%) vessel, and with no difference between RP and nRP.

### 3.2. Surgical, Clinical and Pathological Outcomes

In total, 59/76 (77%) patients underwent pancreatic resection, while in 17/76 (23%) resection was aborted because of intraoperative evidence of non-resectability, according to the surgeon’s judgement. PD was the most frequent operation (44/59, 57.9%), as shown in Table 2.

Biopsies of suspicious perivascular tissues around veins (portal vein, superior mesenteric vein) and arteries (superior mesenteric artery, celiac trunk, hepatic artery) were performed in 32 patients (42.11%), with positive frozen sections in 8/32 (25.0%) cases. In this subgroup of patients with positive frozen sections, we achieved radical resection with AD in six out of eight cases. Vascular resections were performed in 12/76 (15.78%) patients, including superior mesenteric vein (SMV) (N = 7, 58.33%), portal vein (PV) (N = 1, 8.33%), splenomesenteric confluence (N = 1, 8.33%) and celiac trunk (N = 3, 25%). Postoperative complications occurred in 39 patients (51.32%) with Clavien–Dindo ≥ 3b in 8% of cases (6/76). Among the six patients who developed severe postoperative complications (CD ≥ 3), four patients died, resulting in an FTR of 80% and an overall in-hospital mortality of 6.67% (4/59) in the RP. Two of the four patients with in-hospital mortality underwent preoperative chemo-radiotherapy. No patient who underwent venous resection experienced thrombosis. In-hospital mortality in the nRP group was nil.

R1 status was documented in 20/59 patients (33.90%), and 6/59 patients (10.17%) had microscopic vascular infiltration on pathological specimen (Table 3).

### 3.3. Follow-Up and Recurrence Data

Among RP (N = 59), 16 (37.3%) and 6 (10.17%) were able to receive adjuvant chemo- or chemoradiotherapy, respectively. Table 4 shows follow-up data for the RP (N = 59), with 40/59 (67.79%) patients who experienced recurrence, including local (10/40, 25.0%) and systemic recurrence (30/40, 75.0%). Median RFS time was 7 months (5–12, Table 4a). Early recurrence (<6 months) occurred in 16/59 (27.1%) of patients.

Elevated Ca 19,9 serum values after NAT were associated with a lower RFS (HR = 1, 95% CI = 1.00–1.01, *p* value = 0.034) and a shorter PRS (HR: 1.33; 95% CI: 0.99–1.79; *p* = 0.052), suggesting a potential prognostic role. In logistic regression analysis, only induction folfirinox was associated with a trend towards decreased risk of recurrence (OR = 0.143), although this association did not reach statistical significance (*p* = 0.0511).

Among patients who developed recurrence (40/59, 67.79%), the median PRS was 23 months (95% CI: 18–29), with a 1-year PRS rate of 82.5% (95% CI: 66.8–91.3%).

Among non-resected patients, 11/17 (65.71%) received postoperative chemotherapy, and 3/17 (17.56%) received radiotherapy.

### 3.4. Predictors of Resectability

In univariate and multivariate logistic regression analyses, only neoadjuvant folfirinox was associated with higher resection rates (OR = 4.07, 95% CI: 1.14–9.24, *p* = 0.028 and OR = 3.23, 95% CI: 1.59–9.90, *p* = 0.040, respectively) (Supplementary Table S1). Despite a trend suggesting that the majority of patients who underwent preoperative radiotherapy were ultimately resected (29 out of 36, Table 1), logistic regression analyses did not identify a statistically significant association between radiotherapy and resectability. Surprisingly, neither Ca 19,9 nor Ca 19,9 normalization showed any correlation with resectability (Supplementary Table S1).

CT-based evaluation of persisting arterial involvement had low sensitivity and moderate discriminative power. Conversely, all patients without pathological perivascular infiltration, as evaluated with intraoperative frozen sections, were correctly classified as resectable (Supplementary Table S2 and Supplementary Figure S1).

### 3.5. Survival Analysis

**Figure 1:**
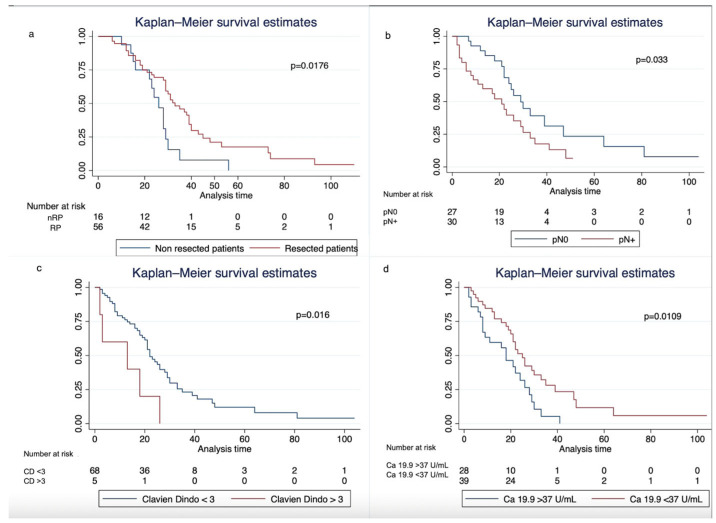
Kaplan–Meier curves of overall survival from diagnosis stratified by resection status (**a**), pN status (**b**), Clavien–Dindo grade 3 complications (**c**) and Ca 19,9 levels normalization after induction therapy (**d**); nRP = non-resected patients, RP = resected patients, CD = Clavien–Dindo.

Median OS from diagnosis was 31 months (95% CI: 29–35) in the entire cohort, 26 months (95% CI: 16–29) in nRP, and 33 months (95% CI: 29–39) in RP (log-rank test *p* = 0.0176, Table 4b, Figure 1a).

Median OS from surgery was 22 months (95% CI: 18–26) in the entire cohort, 25 months (95% CI: 21–30) in RP, and 19 months (95% CI: 9–21) in nRP (log-rank test *p* = 0.0031; Supplementary Figure S2 and Table 4c). The median follow-up from surgery was 21 months (±17.39) and was recorded for 75 of 76 patients. In the entire cohort of patients, early death (<90 days) for disease progression occurred in three of the RP, while no patients died within 90 days in the nRP.

Pathological positive lymph nodes and CD grade > 3b postoperative complications were associated with worse OS, while normalization of Ca 19,9 levels showed correlation with prolonged survival (Figure 1b–d). The Cox proportional hazards model evidenced that RP (HR = 0.39, 95% CI: 0.21–0.75, *p* = 0.005) and patients with Ca 19,9 normalization after induction therapy (HR = 0.56, 95% CI: 0.35–0.88, *p* = 0.014) had a significantly lower risk of death, whereas patients with CD grade ≥ 3b postoperative complications (HR = 2.93, 95% CI: 1.15–7.47, *p* = 0.025), pathological positive lymph nodes (HR = 1.95, 95% CI: 1.03–3.68, *p* = 0.039), higher levels of Ca 19,9 after induction therapy (HR = 1, 95%, *p* = 0.013) and vascular infiltration on pathological specimen (HR = 3.16, 95% CI: 1.21–8.24, *p* = 0.018) showed worse survival outcomes (Table 5).

Even among patients who underwent R1 resection, median OS was significantly longer compared to nRP (26 vs. 19 months; *p* = 0.009), with a 68% relative reduction in mortality risk (HR 0.32, *p* = 0.009).

Surgical resection, pathological nodal involvement, and Ca 19,9 levels < 37 U/mL after NAT remained associated with better survival outcomes when considering survival from diagnosis (Supplementary Table S3). At 12 months from diagnosis, survival was similar between RP and nRP; however, long-term survival at 36 months was markedly higher in the RP (Supplementary Figure S2). Longer duration of chemotherapy (>6 months) was associated with a trend toward better survival outcomes (HR = 0.52, 95% CI: 1.12–151, *p* = 0.085, Supplementary Figure S3). Conditional survival analysis revealed that the estimated probability of surviving an additional 12 months was 73.7% (95% CI: 58.9–83.8%) in RP, compared to 55.6% (95% CI: 26.4–77.2%) in nRP (log-rank test *p* = 0.005, Supplementary Table S4). Recurrence also appeared to impact survival (log-rank *p* = 0.024).

## 4. Discussion

This study evaluates a cohort of patients with borderline resectable or LAPC and arterial involvement at diagnosis, highlighting the potential for a conservative surgical resection following induction therapy and confirming the limitations of current imaging in preoperative assessment.

In our cohort of patients with PDAC and arterial involvement of various degrees, resection after neoadjuvant therapy was associated with a significant survival benefit compared to nRP, with comparable arterial radiological involvement and response to NAT. Furthermore, after recurrence, survival was longer than the overall median survival in the nRP, as if resection per se was of benefit. These findings are consistent with prior studies in LAPC [20,43] and reinforce the importance of surgical resection as part of multimodal treatment strategies for achieving survival benefit, even in cases with BR or LAPC, which harbour a higher risk of positive margins and recurrence [44]. In this context, induction therapies play a pivotal role in selecting patients with favourable baseline characteristics and tumour biology, thereby allowing surgery, when feasible, to deliver meaningful survival benefits. Within this setting, predictors of resectability become critically important. In our series, neoadjuvant folfirinox was independently associated with higher odds of resection, consistent with the growing evidence of its impact on higher resection rate and downstaging in patients with LAPC [45,46].

In line with the previous literature, the accuracy of CT staging and radiological response in predicting resectability and perivascular infiltration was confirmed as a critical issue [47,48]. Indeed, all histologically confirmed vascular infiltrations involved venous structures, with no cases of arterial wall infiltration observed, despite persistent arterial abutment on imaging. CT may overestimate residual vascular involvement, particularly after neoadjuvant therapy. This highlights the role of intraoperative accurate evaluation of real arterial involvement to avoid denying resection in potentially operable cases, while also underscoring the need for improved imaging criteria and multidisciplinary assessment to guide decision-making and surgical planning after induction therapies.

In line with recent reports [49], recurrence was significantly associated with worse OS regardless of its timing, as highlighted by the conditional survival analysis restricted to patients who survived at least 6 months postoperatively. Notably, the median RFS in our cohort was 7 months, which is slightly lower than the RFS values reported in comparable populations of patients with BR or LAPC, where median RFS typically ranges between 9 and 12 months [50]. In this regard, we would underline that RFS is strictly related to the timing of follow-up examinations and definition of recurrence (timing of tests after resection, increase in Ca19,9 only, imaging evidence of relapse), and it is, by nature, not always adherent to the real course of the disease. Overall survival and global quality of life should be the best indicators of a therapeutic approach. Although not statistically significant, elevated post-induction CA 19,9 levels showed a trend toward association with shorter post-recurrence survival. This is consistent with prior studies linking tumour marker response to prognosis and supports its potential value in risk stratification after recurrence.

### 4.1. The Role of Radiotherapy in the Induction Strategy

In the present series 36 out of 76 patients received chemotherapy followed by radiochemotherapy as a full course of neoadjuvant therapies. A trend toward a better outcome in the subgroup of patients who received the full course of preoperative therapies in terms of resectability is insufficient to support the routine use of radiotherapy in this context. Furthermore, the two patients who died from surgical complications had also received radiotherapy, which could make the steps of tumour resection more difficult, resulting in more severe complications [51]. Further well-designed RCTs on radiotherapy in this subset would clarify the real impact of this approach [52,53].

### 4.2. Surgical Decision-Making in Persistent Perivascular Infiltration After Induction Therapy

Surgery for LAPC after induction therapy remains a challenging issue. In this scenario, different management options can be considered: definitive palliation chemoradiotherapy, surgical exploration with potential vascular resection, or surgical resection without formal vascular resection [30,54]. While our study has confirmed the survival benefit of surgery compared to palliative strategies in a homogeneous cohort of patient responders to induction therapies deemed as potentially resectable [15], the optimal surgical approach for tumours with arterial involvement remains controversial. Although the surgical approach was not applied through a formal protocol, the consistent avoidance of AR on the SMA—due to the high burden of postoperative morbidity and mortality and the potential detrimental impact on quality of life—together with the growing experience in AD techniques, allowed our team to refine both patient selection and intraoperative judgement over time. This systematic conservative approach, which relies on adopting AD and using intraoperative frozen section analysis to evaluate suspicious tissue surrounding arteries, allowed us to achieve survival and post-recurrence data similar to that reported in other series of surgically treated LAPC with AR [14,18,55,56].

These findings suggest that, in selected patients, AD may offer oncologic outcomes similar to more extensive ARs, potentially reducing perioperative risks and preserving postoperative quality of life. Although recent reports from expert centres have demonstrated promising results with extended resections involving the SMA [56,57], their application remains limited due to technical complexity and historical concerns regarding long-term oncological benefit. Additionally, histological studies have demonstrated that the rate of true infiltration of the resected arterial segment is around 30–45% [13,14,15]. Therefore, it remains unclear whether the small number of patients who survived longer after AR belonged to the subgroup with confirmed histological infiltration. Whether patients in our cohort deemed unresectable after surgical exploration and treated with palliative chemotherapy could have benefited from a more aggressive surgical approach, including AR, remains an open question. The median OS in this group was limited to 19 months, which appears modest when compared to the more favourable outcomes reported in selected series of AR [13]. These findings raise the possibility that, in carefully selected cases not suitable for AD alone, AR might offer a survival benefit.

Finally, in our series of RPs, three patients experienced of early death due to rapid tumour progression, irrespective of preoperative marker normalization, radiological response and negative tumour margins. Despite the limitations of the small sample size, this finding underscores the need for more accurate patient stratification and better characterization of disease biology to avoid ‘futile’ surgery.

Further research is needed to clarify the role of surgical resection in LAPC; to better define the utility of frozen sections, which in our series showed a risk of false negatives, particularly when sampling fibrotic perivascular tissue [57]; and to establish reliable markers of biologically aggressive disease (e.g., liquid biopsy with determination of tumour circulating DNA [58]) for which surgery may be futile, even when technically feasible.

### 4.3. Limits

The main limitation of the study is its observational and retrospective design.

Despite allowing for the reproducibility of surgical techniques and preoperative and postoperative management, the monocentric design limited the cohort of patients, thus reducing the power of statistical analysis. Being a surgical series, we acknowledge the intrinsic limit of our study compared to “real-life observational” ones since the entire cohort of patients only included those who had a good-quality imaging, necessary to classify resectability status, and who ultimately were considered eligible for surgical exploration, omitting the whole cohort of patients diagnosed with locally advanced PDAC.

## 5. Conclusions

A conservative surgical approach based on AD in patients with LAPC represents a valuable technique associated with acceptable outcomes. A selective multimodal approach, guided by accurate patient selection, response to induction therapy and surgical expertise, remains prudent and oncologically valid in a context wherein extensive vascular resections are still not validated as curative therapies and there are concerns related to quality of life. Future prospective studies, comparing patients undergoing AD versus AR, with histopathologic assessment of true invasion of resected arteries and its correlation with survival and quality of life, will help to clarify the real benefit of each strategy and define more precise criteria for patient selection.

## Figures and Tables

**Table 1:** Baseline characteristics of the entire cohort.

	Total No (N = 76)	Resected (N = 59)	Non-Resected (N = 17)	*p*-Value
Female Sex, N (%)	38 (50%)	29 (49.15%)	9 (52.94%)	0.783
Age, median (IQR)	62 (53.5–68.5)	61 (53–68)	65 (61–71)	0.184
BMI	23.1 (21.22–26.7)	23.095 (21.09–26.19)	24.91 (22.03–27.18)	0.217
Diabetes	15 (19.74%)	11 (18.64%)	4 (26.67%)	0.357
Jaundice	41 (53.95%)	32 (54.24%)	9 (56.25%)	0.886
Ca 19,9 at diagnosis	206.35 (80.3–618.5)	218.95 (83.35–704.75)	117.5 (75.4–343)	0.511
Ca 19,9 preoperative	25.2 (13.5–71.7)	23.5 (12.6–65.1)	37 (18.2–72.9)	0.257
ΔCA 19,9 ≥ 50%	39 (51.31%)	34 (57.62%)	5 (29.41%)	0.453
CEA at diagnosis	2.55 (1.6–4.6)	2.6 (1.6–4.6)	2.3 (1.3–4.6)	0.591
Tumour location				
*Head*	46 (60.52%)	37 (62.71%)	9 (56.25%)	0.868
*Uncinate process*	13 (17.10%)	10 (16.95%)	3 (18.75%)	
*Isthmus*	6 (7.89%)	4 (6.78%)	2 (12.50%)	
*Body–tail*	10 (13.16%)	8 (13.56%)	2 (12.50%)	
Tumour shrinkage, median (mm)	9 (0–19)	9.5 (0–17.5)	9 (3–22)	0.850
Resectability at diagnosis				
*BR*	53 (69.74%)	42 (71.19%)	11 (64.71%)	0.608
*LA*	23 (30.26%)	17 (28.81%)	6 (35.29%)	
NAT regimen				
*Folfirinox*	46 (60.53%)	40 (68.97%)	6 (35.29%)	0.013
*Folfirinox + Gem-NabP*	1 (1.32%)	1 (1.72%)	0 (0%)	
*Gem + Nab-P*	18 (23.68%)	12 (20.69%)	6 (35.29%)	
*Gemcitabine alone*	5 (6.8%)	1 (1.72%)	4 (23.53%)	
*Gemox*	2 (2.63%)	2 (3.45%)	0 (0%)	
*PAX-G*	2 (2.63%)	1 (1.72%)	1 (5.88%)	
Other	1 (1.32%)	1 (1.72%)	0 (0%)	
NAT cycles (n)	8 (4–14)	6 (4–14)	11 (4–14)	0.850
NAT duration (months)	8 (6–9)	8 (6–9)	8 (5–10)	0.845
Neoadjuvant RT	36 (47.37%)	29 (49.15%)	7 (41.18%)	0.562
T/A interface at restaging				
*No contact*	9 (11.84%)	9 (15.25%)	0 (0%)	0.339
*Abutment or encasement*	67 (88.16%)	50 (84.74%)	17 (100%)	
Any arterial involvement after NAT	67 (88.16%)	50 (84.74%)	17 (100.00%)	0.118
*SMA*	40 (59.70%)	33 (66%)	7 (41.18%)	0.167
*HA*	19 (28.36%)	12 (24%)	7 (41.18%)	
*Celiac trunk*	8 (11.94%)	5 (10%)	3 (17.65%)	
Ca 19,9 post NAT	25.2 (13.5–71.7)	23.5 (12.6–65.1)	37 (18.2–72.9)	0.257
Radiological response after induction therapies				
*Stable disease*	55 (72.37%)	40 (67.80%)	15 (88.24%)	0.084
*Partial response*	21 (27.63%)	19 (32.20%)	2 (11.76%)	

Notes: Continuous variables are presented as median (IQR); categorical variables as n (%). No: Number; BMI: body mass index; BR: borderline resectable; LA: locally advanced; SMA: superior mesenteric artery; HA: hepatic artery; T/A interface: tumour/artery interface; NAT: neoadjuvant chemotherapy; Pax-G: cisplatin, nab-paclitaxel, capecitabine and gemcitabine; Gem-Nab-P: gemcitabine (GEM) plus nab-paclitaxel (NabP); GEMOX: gemcitabine and oxaliplatin; folfirinox: folinic acid, fluorouracil, irinotecan, oxaliplatin.

**Table 2:** Surgical and postoperative data of the entire cohort.

	Total No (N = 76)	RP	nRP
Type of Surgery			
*Pylorus-preserving pancreaticoduodenectomy*	34 (44.74%)	34 (57.62%)	0 (0%)
*Whipple pancreaticoduodenectomy*	10 (13.16%)	10 (16.94%)	0 (0%)
*Distal pancreatectomy*	8 (10.53%)	8 (13.56%)	0 (0%)
*Total pancreatectomy*	4 (5.26%)	4 (6.78%)	0 (0%)
*Distal spleen pancreatectomy with celiac axis resection*	3 (3.95%)	3 (5.08%)	0 (0%)
*Exploratory laparotomy without resection*	17 (22.36%)	0 (0%)	17 (100%)
Frozen sections of perivascular biopsy	32 (42.11%)	28 (47.46)	4 (23.53%)
*Positive*	8 (25.00%)	6 (21.43%)	2 (50.00%)
*Negative*	24 (75.00%)	22 (78.57%)	2 (50.00%)
Perivascular biopsy	32 (40.78%)	28 (47.45%)	4 (23.53%)
*SMV*	10 (31.25%)	10 (16.95%)	0 (0.00%)
*PV*	2 (6.25%)	2 (3.39%)	0 (0.00%)
*SMA*	6 (18.75%)	5 (8.47%)	1 (5.88%)
*HA*	10 (31.25%)	7 (11.86%)	3 (17.65%)
*Celiac trunk*	3 (9.38%)	3 (5.08%)	0 (0.00%)
*Other*	1 (3.12%)	1 (1.69%)	0 (0.00%)
Intraoperative blood loss (mL)	350 (150–600)	400 (250–600)	100 (0–220)
Surgery duration (min)	375 (257.5–452.5)	400 (355–500)	160 (70–205)
Vascular resections	12 (15.79%)	12 (20.34%)	0 (0.00%)
*SMV*	7 (58.33%)	7 (11.86%)	
*PV*	1 (8.33%)	1 (1.69%)	
*PV confluence*	1 (8.33%)	1 (1.68%)	
*Celiac trunk*	3 (25%)	3 (5.08%)	
Postoperative complications	39 (51.32%)	35 (59.32%)	4 (23.53%)
Clavien–Dindo ≥ 3	6 (8.00%)	5 (8.47%)	1 (5.88%)
POPF	10 (13.16%)	10 (16.95%)	0 (0.00%)
*BL*	5 (6.58%)	5 (8.47%)	
*Grade B/C*	5 (6.58%)	5 (8.47%)	
Abdominal fluid collection	7 (9.21%)	7 (11.86%)	0 (0.00%)
Biliary fistula	3 (3.95%)	3 (5.08%)	0 (0.00%)
Enteric leak	0 (0%)	0 (0.00%)	0 (0.00%)
Chylous fistula	7 (9.21%)	7 (11.86%)	0 (0.00%)
PPAP	4 (5.26%)	4 (6.78%)	0 (0.00%)
PPH	6 (7.89%)	4 (6.78%)	2 (11.76%)
DGE	3 (3.95%)	3 (5.08%)	0 (0.00%)
Reintervention	5 (6.58%)	4 (6.78%)	1 (5.88%)
In-hospital death	4 (5.26%)	4 (6.67%)	0 (0.00%)
Readmission	6 (7.89%)	6 (10.17%)	0 (0.00%)
FTR	4/6 (66.66%)	4/5 (80%)	0/1 (0%)

Notes: Continuous variables are presented as median (IQR); categorical variables as n (%). No: number; min: minutes; mL: millilitres; RP: resected patients; nRP: non-resected patients; SMA: superior mesenteric artery; HA: hepatic artery; PV: portal vein; SMV: superior mesenteric vein; POPF: postoperative pancreatic fistula; PPAH: post-pancreatectomy acute pancreatitis (PPAP); PPH: post-pancreatectomy hemorrhage; DGE: delayed gastric emptying. FTR = failure to rescue rate, calculated as the proportion of patients who died among those who experienced a severe postoperative complication.

**Table 3:** Pathological outcomes of the resected patients (N = 59).

	Resected Patients (N = 59)
Grading	
*G1*	20 (33.89%)
*G2*	36 (61.02%)
*G3*	3 (35.08%)
T status	
*T0*	3 (5.08%)
*T1*	17 (28.81%)
*T2*	22 (37.29%)
*T3*	15 (25.42%)
*T4*	2 (3.39%)
Nodal Status	
pN1-pN2	32 (54.24%)
pN0	27 (46.76%)
R status	
*R0*	39 (66.10%)
*R1*	20 (33.90%)
Positive retroperitoneal margin	13 (22.03%)
Positive SMV groove	7 (11.86%)
Microscopic vascular infiltration	6 (10.17%)
*SMV*	6 (85.71%)
*PV*	1 (14.29%)
CAP	
Not reported	27 (45.76%)
0	2 (3.39%)
1	6 (10.17%)
2	17 (28.81%)
3	7 (11.86%)
Perineural invasion	43 (72.88%)

Notes: Categorical variables are expressed as n (%). SMA: superior mesenteric artery; HA: hepatic artery; SMV: superior mesenteric vein; GDA: gastroduodenal artery; PV: portal vein; CAP: College of American Pathologists tumour response scoring system.

**Table 4:** Recurrence analysis of resected patients (RP); RFS: recurrence-free survival; 95% CI: 95% confidence intervals (a); survival analysis from diagnosis of resected (RP) and non-resected patients (nRP); CI: 95% confidence intervals (b); survival analysis from surgery of resected and non-resected patients; CI: 95% confidence intervals (c).

(**a**)					
	**1-Year RFS (%)**	**95% CI**	**3-Year RFS (%)**	**95% CI**	**Median RFS, Months (95% CI)**
Resected patients	22.2%	10.5–36.7%	2.78%	0.21–12.37%	7 (5–12)
(**b**)					
**1-Year OS (%)**	**95% CI**	**3-Year OS (%)**	**95% CI**	**Median OS, Months**	**(95% CI)**
RP	94.64%	84.30–98.24%	45.73%	31.50–58.87%	33 (29–39)
nRP	93.75%	63.23–99.10%	7.79%	0.49–29.36%	26 (16–29)
(**c**)					
	**1-Year OS (%)**	**95% CI**	**3-Years OS (%)**	**95% CI**	**Median OS, Months (95% CI)**
RP	75.44%	62.08–84.65%	29.72%%	17.19–43.34%	25 (21–30)
nRP	73.66%	44.07–89.22%	-	-	19 (9–21)

**Table 5:** Analysis of overall survival (Cox proportional hazards model). HR = hazard ratio (HR); BR = borderline resectable; LA = locally advanced; NAT: neoadjuvant therapy; IC 95% = 95% confidence interval. Bold were used for significant *p* values.

Variable	HR	IC95%	*p*-Value	Comment
Surgical resection	0.39	0.21–0.75	**0.005**	Resection is associated with improved survival outcomes, with resected patients having 61% lower risk of death compared to non-resected patients.
Pathological positive lymph nodes	1.95	1.03–3.68	**0.039**	Patients with pathological positive lymph nodes showed worse survival outcomes.
Clavien–Dindo ≥ 3	2.93	1.15–7.47	**0.025**	Postoperative complications significantly worsen survival outcomes.
Radiological response	1.12	0.63–2.00	0.667	
BR/LA status post NAT	1.10	0.74–1.63	0.631	
Normal Ca 19,9 after NAT	0.56	0.35–0.88	**0.014**	Ca 19,9 serum level normalization after induction therapy is associated with improved survival outcomes (44% lower risk of death).
Abnormal Ca 19,9 after NAT	1.003	1.00–1.00	**0.013**	Increased levels of Ca 19,9 after induction therapy are associated with worse survival outcomes.
Vascular infiltration at histology	3.16	1.21–8.24	**0.018**	
Recurrence	4.81	1.14–20.20	**0.032**	Recurrence significantly worsens survival outcomes.

## Data Availability

The data presented in this study are available on request from the corresponding author.

## Supplementary Materials

The following supporting information can be downloaded at: https://www.mdpi.com/article/10.3390/cancers18050830/s1, Figure S1: ROC curve showing the diagnostic accuracy of preoperative CT imaging for detecting perivascular tumour infiltration through intraoperative frozen section; Figure S2: Kaplan–Meier curves of survival from surgery stratified by surgical resection; Figure S3: Kaplan–Meier curves of survival from diagnosis stratified by number of induction therapy cycles; Table S1: Univariable and Multivariable Logistic Regression Analyses for predicting resectability; Table S2: Accuracy of computed tomography (CT)-based evaluation of persisting arterial involvement after induction therapy in predicting perivascular infiltration on final pathology; Table S3: Survival from diagnosis outcomes by surgical resection status and duration of induction chemotherapy; Table S4: Conditional survival analysis using the Kaplan–Meier method and Cox proportional hazards model.
