# Supplementary material for: The Role of a “Conservative” Resection Strategy After Neoadjuvant Treatment for Borderline/Locally Advanced PDAC with Arterial Involvement: A Single-Centre Retrospective Observational Study
Source: Cancers (Basel). 2026 Mar 4;18(5):830. doi: 10.3390/cancers18050830 (PMC12984494; doi:10.3390/cancers18050830)
Supplement: Supplementary file 1 [file cancers-18-00830-s001.zip › SUPPLEMENTARY/supplementary.pdf]

Table S1. Univariable and Multivariable Logistic Regression Analyses for Predicting Resectability.				
**Variable**	**Univariable OR (95% CI)**	**p-value**	**Multivariable OR (95% CI)**	**p-value**
Age	0.95 (0.90 - 1.02)	0.186	0.96 (0.90-1.03)	0.285
Sex	0.85 (0.29 - 2.53)	0.783		
Ca 19.9 before NAT	1 (0.99- 1.00)	0.558		
Ca 19.9 after NAT	0.99 (0.99- 1.00)	0.952	1.00 (0.99-1.01)	0.410
Ca 19.9 normalization after induction therapy				
Normal Ca 19.9 after NAT	1.85 (0.64-4.74)	0.90	2.40(0.64-9.03)	0.193
N status at CT	0.26 (0.077 - 0.93)	**0.039**		
Tumor location	1.11 (.71- 1.75)	0.639		
Neoadjuvant FOLFIRINOX	4.07 (1.14 - 9.24)	**0.028**	3.23 (1.05 - 9.90)	**0.040**
Neoadjuvant radiotherapy	1.38 (0.46 - 4.12)	0.563	2.44 (0.68-8.70)	0.169
NAT cycles	0.96 (0.85- 1.10)	0.645		
Radiological response	0.28 (0.058- 1.35)	0.113		

Table S2: Accuracy of Computed Tomography (CT) – based evaluation of persisting arterial involvement after induction therapy in predicting peri-vascular infiltration on final pathology. PPV: positive predictive value; NPV: negative predictive value; AUC: Area Under the Curve.
The area under the ROC curve (AUC) was estimated both via non-parametric ROC analysis (AUC = 0.58) and as the average of sensitivity and specificity (AUC = 0.64).

Outcome Predicted	Sensitivity (%)	Specificity (%)	PPV (%)	NPV (%)	AUC (95% CI)
Peri - vascular infiltration on pathological specimen	28.6%	100%	28.6%	100%	0.64 (0.56- 0.73)

Table S3. Survival from diagnosis outcomes by surgical resection status and duration of induction chemotherapy. OS: overall survival; HR: hazard ratio from Cox model. 95% CI= 95% confidence intervals.

**Variable**	**Group**	**12-month**	**36-month survival**	**Median survival**	**Log-rank p**	**HR (95% CI)**	**p (Cox)**

				in months (95%CI)			
Surgical Resection:	*Non-resected*	93.75%	7.79%	26 (16- 29)	0.0176	0.48 (0.25-0.89)	**0.022**
	Resected	89.29%	45.88%	33 (29-39)			
Induction chemotherapy	*≤ 6 months*	60.00%	15.00%	14 (6-32)	0.9759	0.52 (1.12 - 151)	0.095
	>6 months	95.83%	48.90%	35 (28-40)			
Ca 19,9 levels normalization after induction therapy:<br>Yes<br>Not	*Ca 19,9 <37U/mL*	94.87%	44.92%	32 (29-40)	0.0406	0.58 (0.37 – 0.92)	**0.020**
	Ca 19.9 > 37 U/mL	80.77%	27.66 %	26 (23-)			
Pathological nodal involvement	*Yes*	89.66%	38.93%	28 (16-39)	0.02	2.02 (1.08-3.78)	0.027
	No	100%	52.82%	39 (31-54)			

Table S4: conditional survival analysis using the Kaplan–Meier method and Cox proportional hazards model. 95% CI= 95% confidence intervals. HR= Hazard ratio.

**Time Point**	**Variable**	**Group**	**Conditional Survival (%)**	**95% CI**	**p-value (log-rank)**
After 12 months	Recurrence	No Recurrence	100.00%	-	
		Recurrence	86.49%	70.53% – 84.10%	**0.024**
		HR (Cox)	HR = 2.01e+16	-	**1**
After 12 months	Surgical resection	Resected	84.31%	71.07% – 91.08%	
		Not resected	80.00%	49.98% – 93.00%	**0.0005**
		HR (Cox)	0.31 (0.16 – 0.63)		**0.001**

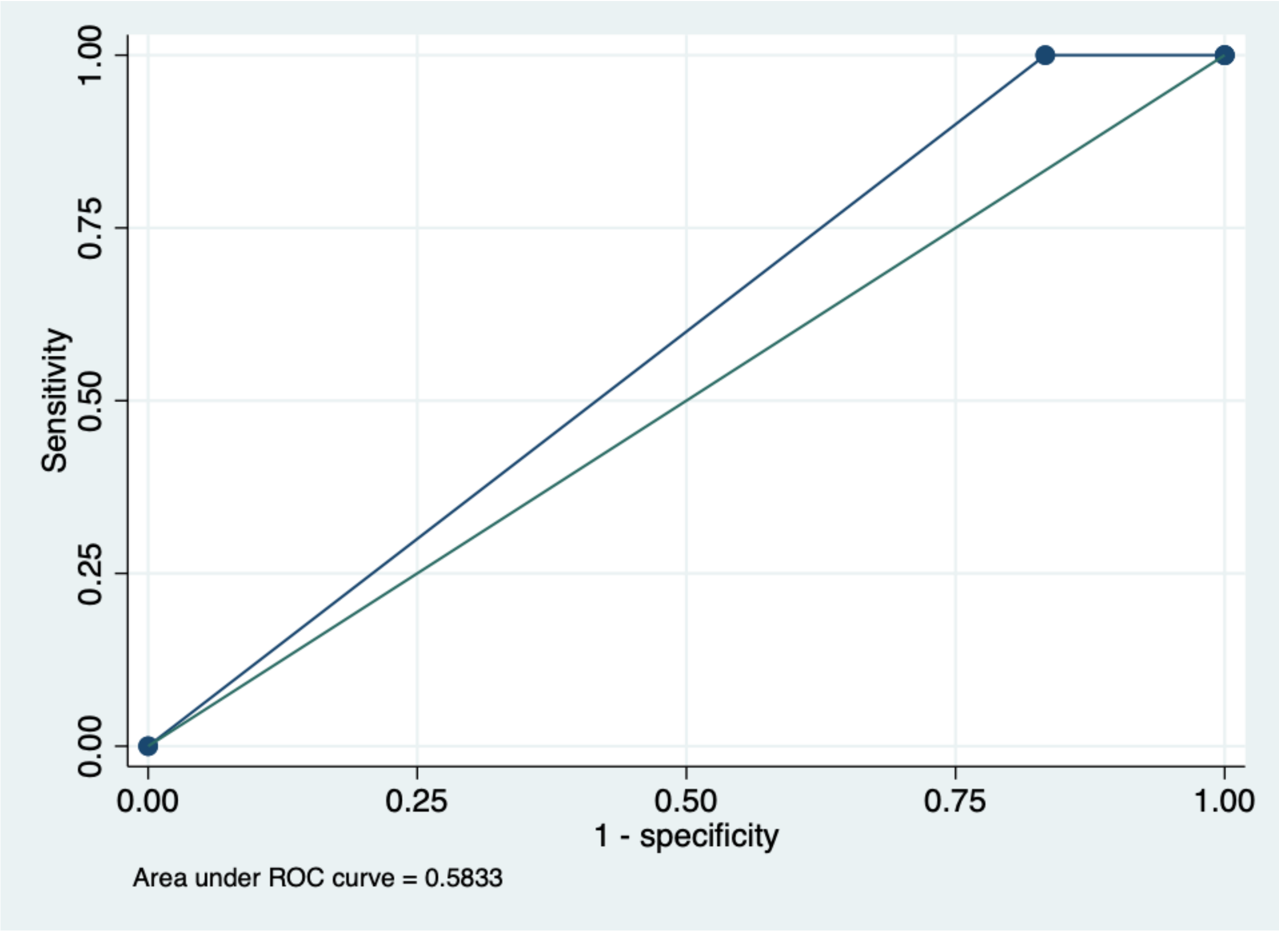

Figure S1: ROC curve showing the diagnostic accuracy of preoperative CT imaging for detecting perivascular tumor infiltration through intraoperative frozen section. Blue ROC curve refers to the diagnostic performance of CT classification. Green diagonal line refers to the line of no discrimination (AUC = 0.5)

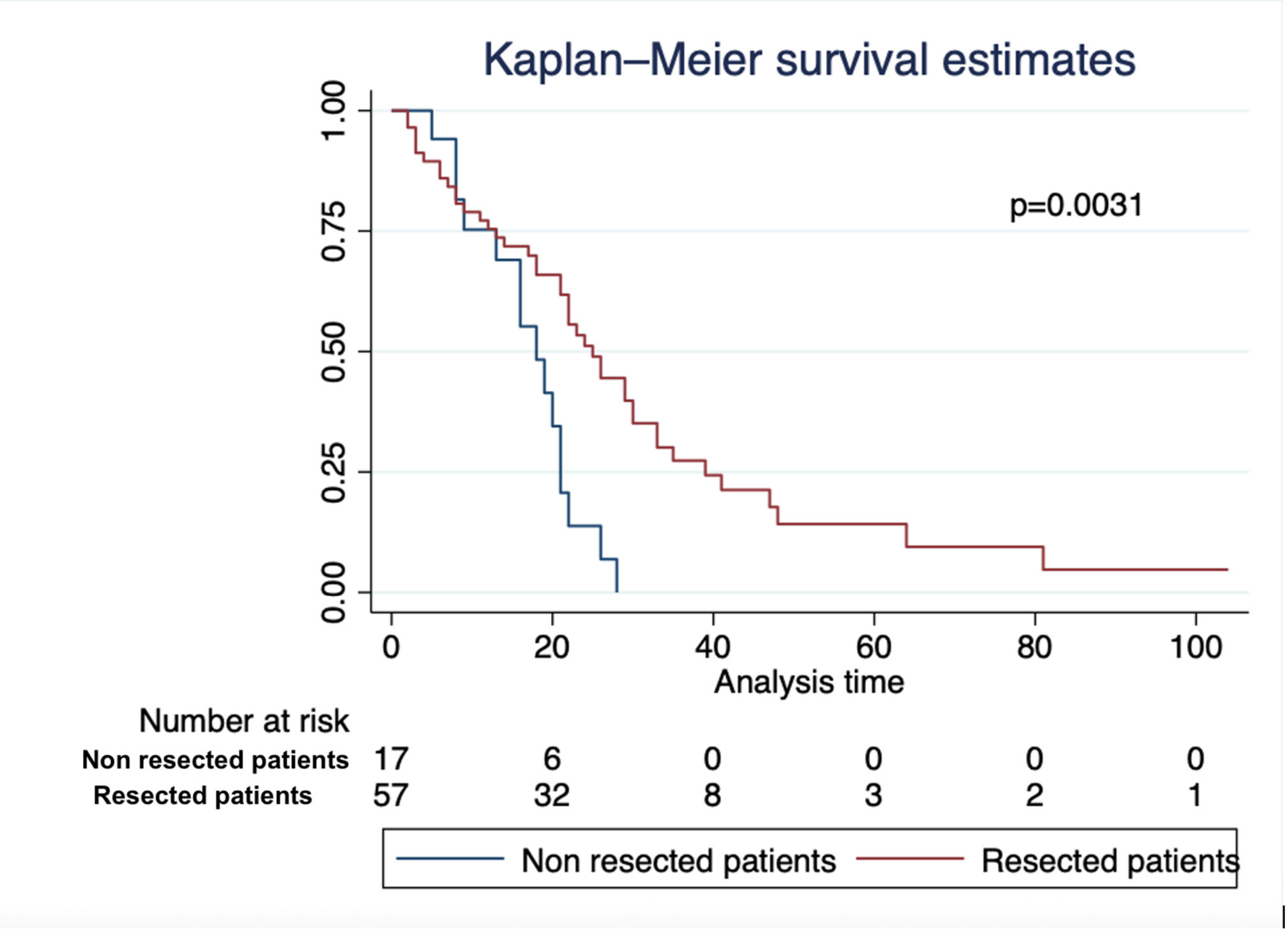

Figure S2: Kaplan–Meier curves of survival from surgery stratified by surgical resection: the difference in survival between the two groups was statistically significant (log-rank test, p = 0.0031).

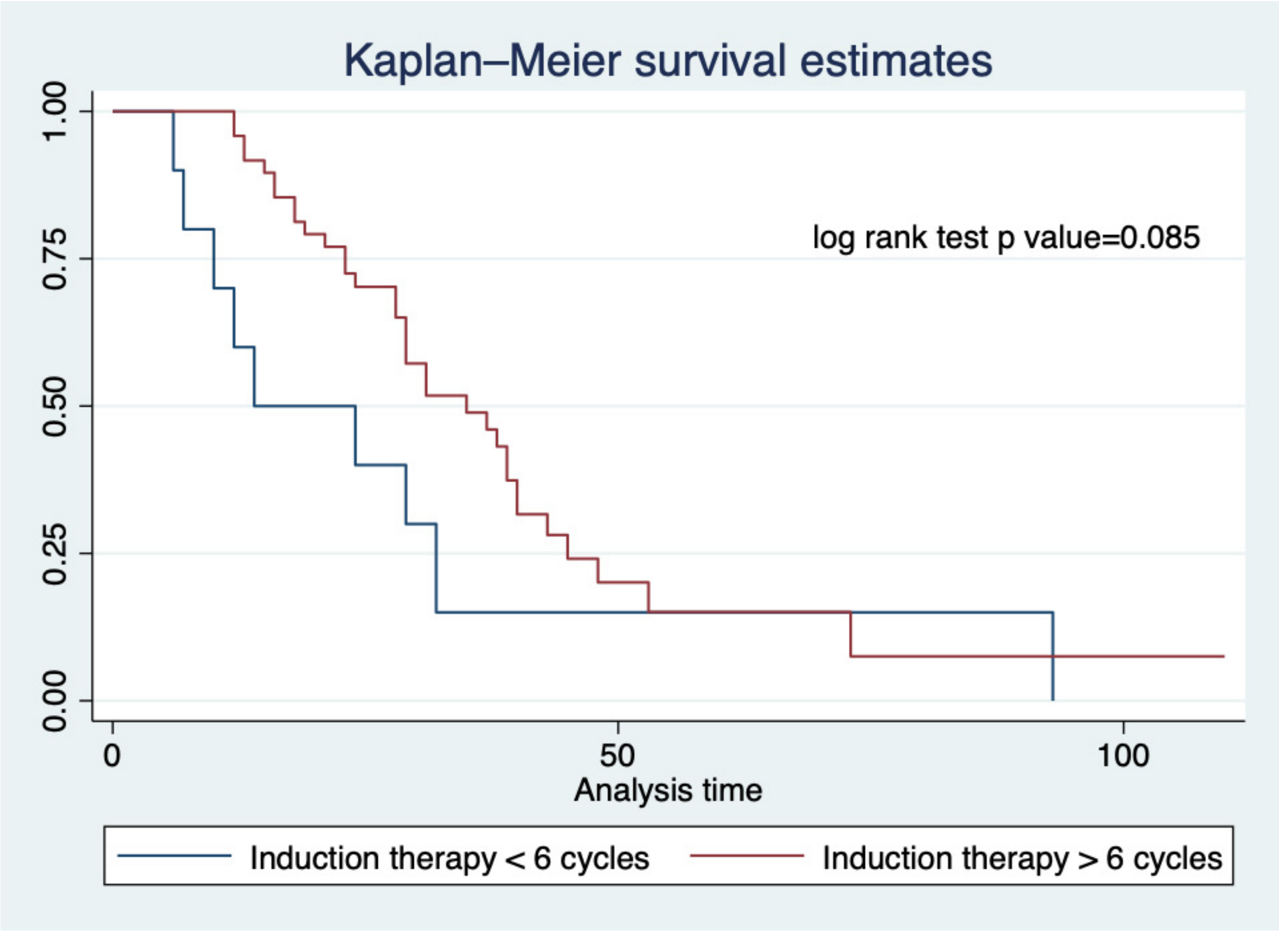

Figure S3: Kaplan–Meier curves of survival from diagnosis stratified by number of induction therapy cycles: a trend towards significance was observed with improved survival for patients who received at least 6 cycles of induction therapy (log-rank test, $p = 0.085$).
